# https://invertebratefungi.org/: an expert-curated web-based platform for the identification and classification of invertebrate-associated fungi and fungus-like organisms

**DOI:** 10.1093/database/baac021

**Published:** 2022-04-01

**Authors:** De-Ping Wei, Eleni Gentekaki, Kevin D Hyde, Yuan-Pin Xiao, Thatsanee Luangharn, Qing Tian, Yuan-Bing Wang, Saisamorn Lumyong

**Affiliations:** CAS Key Laboratory for Plant Diversity and Biogeography of East Asia, Kunming Institute of Botany, Chinese Academy of Sciences, Panlong District, Kunming, Yunnan, P.R. 650201, China; Center of Excellence in Fungal Research (CEFR), Mae Fah Luang University, Muang District, Chiang Rai 57100, Thailand; Department of Entomology and Plant Pathology, Faculty of Agriculture, Chiang Mai University, Mueang Chiang Mai, Chiang Mai 50200, Thailand; Center of Excellence in Fungal Research (CEFR), Mae Fah Luang University, Muang District, Chiang Rai 57100, Thailand; School of Science, Mae Fah Luang University, Muang District, Chiang Rai 57100, Thailand; CAS Key Laboratory for Plant Diversity and Biogeography of East Asia, Kunming Institute of Botany, Chinese Academy of Sciences, Panlong District, Kunming, Yunnan, P.R. 650201, China; Center of Excellence in Fungal Research (CEFR), Mae Fah Luang University, Muang District, Chiang Rai 57100, Thailand; Innovative Institute of Plant Health, Zhongkai University of Agriculture and Engineering, Haizhu District, Guangzhou 510225, P.R. China; Research Center of Microbial Diversity and Sustainable Utilization, Faculty of Science, Chiang Mai University, Mueang Chiang Mai, Chiang Mai 50200, Thailand; Center of Excellence in Fungal Research (CEFR), Mae Fah Luang University, Muang District, Chiang Rai 57100, Thailand; School of Science, Mae Fah Luang University, Muang District, Chiang Rai 57100, Thailand; Center of Excellence in Fungal Research (CEFR), Mae Fah Luang University, Muang District, Chiang Rai 57100, Thailand; Center of Excellence in Fungal Research (CEFR), Mae Fah Luang University, Muang District, Chiang Rai 57100, Thailand; School of Science, Mae Fah Luang University, Muang District, Chiang Rai 57100, Thailand; The International Joint Research Center for Sustainable Utilization of Cordyceps Bioresources in China and Southeast Asia, Yunnan University, Wuhua District, Yunnan, P.R. 650091, China; Department of Biology, Faculty of Science, Chiang Mai University, Mueang Chiang Mai, Chiang Mai 50200, Thailand; Research Center of Microbial Diversity and Sustainable Utilization, Faculty of Science, Chiang Mai University, Mueang Chiang Mai, Chiang Mai 50200, Thailand; Academy of Science, The Royal Society of Thailand, Dusit District, Bangkok 10300, Thailand

## Abstract

Fungi are the major decomposers in terrestrial and aquatic ecosystems, playing essential roles in biogeochemical cycles and food webs. The Fungi kingdom encompasses a diverse array of taxa that often form intimate relationships with other organisms, including plants, insects, algae, cyanobacteria and even other fungi. Fungal parasites of insects are known as entomopathogenic fungi and are the causative agents of serious disease and/or mortality of their hosts. Entomopathogens produce distinct metabolic compounds with roles in pathogenicity, virulence and host–parasite interactions. Thus, the potential of discovering new bioactive compounds useful in biocontrol and pharmaceutical industries is high. Given the significance of entomopathogenic fungi, the rapid research advances and the increased interest, it has become necessary to organize all available and incoming data. The website https://invertebratefungi.org/ has been developed to serve this purpose by gathering and updating entomopathogenic genera/species information. Notes of entomopathogenic genera will be provided with emphasis on their taxonomic status. Information on other invertebrates, such as rotifers, will also be included. Descriptions, photographic plates, information on distribution and host (where applicable) along with molecular data and other interesting details will also be provided. The website is easily and freely accessible to users. Instructions concerning the platform architecture and functionality of the website are introduced herein. The platform is currently being expanded and will be continuously updated as part of the effort to enrich knowledge on this group of fungi.

**Database URL**: https://invertebratefungi.org/

## Introduction

Invertebrate organisms account for a major part of animal diversity. Over 97% of all described animal species are invertebrates being omnipresent in terrestrial and aquatic ecosystems (1). Fungi have the ability to colonize a wide range of invertebrate hosts, ranging from soil-inhabiting microinvertebrates to macroinvertebrates in Insecta. Fungi can live as entomopathogens, either endoparasites or ectoparasites, in order to exploit their invertebrate hosts and obtain nutrients (7–11). Invertebrates are known to harbor a vast number of fungi and fungus-like organisms (2). The most available literature on invertebrate fungi focuses on entomopathogens. The taxonomic framework of entomopathogenic fungi has undergone several revisions (2–6). Phylogenetic analyses have shown that entomopathogenic fungi are distributed across six major groups: Ascomycota, Basidiomycota, Chytridiomycota, Microsporidia, zygomycetes and fungus-like Oomycota (2, 5, 6).

The phylum Ascomycota contains high diversity of entomopathogenic fungi, which mainly parasite arthropod hosts in forests and soil-dwelling microinvertebrates such as nematodes and rotifers (9, 12–16). All members of the order Laboulbeniales (Ascomycota) are obligate ectoparasites of the integument of insects, but the effect on their hosts is not lethal (17, 18). Entomopathogenic Basidiomycota comprises the termite egg-infecting genus *Fibularhizoctonia* and the scale insect-parasitic genera *Uredinella* and *Septobasidium* (2). Chytridiomycota includes the entomopathogenic genera *Myiophagus*, *Coelomycidium*, *Myrmicinosporidium* and *Coelomomyces* (2). These infect mostly Diptera hosts and occasionally scale insects (2). Microsporidia is a group of intracellular pathogenic fungi, consisting of 143 genera, 69 of which are known to infect a broad array of insect hosts spanning 12 orders (2). Zygomycetes along with Trichomycetes were initially accommodated in the phylum Zygomycota (19). However, recent phylogenetic analyses have revealed the polyphyletic nature of Zygomycota and, as a consequence, this phylum has now been abandoned and replaced with multiple new phyla (20–26). Entomophthoromycota and Mucoromycota are two of the newly erected Zygomycetes phyla containing entomopathogenic fungi. In Entomophthoromycota, entomopathogens are distributed across 19 genera forming biotrophic associations across 10 orders of insects (2). Mucoromycota contains a large number of Trichomycetes fungi, which are obligate symbionts of the gut of various arthropod larvae in aquatic environments (2). Several genera in Oomycota, namely *Aphanomyces*, *Couchia*, *Crypticola*, *Lagenidium*, *Leptolegnia* and *Pythium*, have been recorded as pathogens of mosquito, midge, lobster and shrimp (2, 27–29).

Invertebrate-associated fungi are highly diverse, with entomopathogens comprising a major constituent of this group. Entomopathogenic fungi play important roles in keeping populations of insects under control in nature and thus have vast potential as biocontrol agents. They also constitute a rich resource of naturally produced bioactive compounds. Many biological insecticides have been developed from this group of fungi and used in protecting crops from pests (30–32). A large variety of secondary metabolites produced by entomopathogenic fungi have been validated as nutraceuticals and traditional remedies (33). However, only a fraction of entomopathogenic species has been successfully exploited for their biocontrol and pharmaceutical value potential. Informal identification or improper taxonomic placement of these fungi is a frequent issue due to the dynamic nature of fungal classification system. This may lead to increasing taxonomic confusion among scientists and mycologists. Therefore, it is essential to provide their corrected fungal names accompanied by information about the biology, host range, distribution and specific functions (34). The accurate identification and precise scientific documentation of these fungi not only benefits communication with mycologists and interested amateurs but also paves the way for downstream utilization, including pharmaceutical industry and biological control applications.

To accumulate and organize available data in a single repository and to effectively communicate the knowledge of fungi to the public, many databases have been designed for different fungal groups, such as Basidiomycota (35), Botryosphaeriales (36), Coelomycetes (https://www.coelomycetes.org/), Dothideomycetes (37), fresh water fungi (38), phytopathogens (39) and Sordariomycetes (40). In addition, notes on changes and additions to phyla, classes, orders, families and genera of fungi are provided on the website Outlineoffungi.org (https://www.outlineoffungi.org/). However, a database dedicated to fungi and fungi-like organisms associated with invertebrates has not yet been developed. The website invertebratefungi.org is therefore established as part of the project titled ‘The future of specialist fungi in a changing climate: baseline data for generalist and specialist fungi associated with ants, *Rhododendron* species and *Dracaena* species’. The aims of this website are to: (i) collect and curate morphological and molecular data on invertebrate-associated fungi from formally published articles and (ii) provide a continuously updated checklist of entomopathogenic genera/species.

### The need for a database of invertebrate fungi

In the past decade, the application of molecular tools has led to a dramatic revolution in the taxonomy of fungal entomopathogens. Molecular analyses have revealed several cases where phenotypic features do not necessarily reflect phylogenetic results (12, 14). A distinct example comes from clavicipitaceous fungi. Several subgenera under Cordyceps sensu lato (Clavicipitaceae) were initially recognized on the basis of the arrangement of perithecia, the disarticulation of ascospores and host affiliation (41–44). Later works revealed that the aforementioned characteristics presented cases of convergent evolution in Clavipitaceae sensu lato (12). Consequently, Clavicipitaceae sensu stricto, Cordycipitaceae and Ophiocordycipitaceae were erected to accommodate species of Cordyceps s. l. and other clavicipitaceous fungi. Combination of morphological and phylogenetic analyses has been crucial for resolving numerous taxonomic issues and for providing a reliable, more robust taxonomic framework of entomopathogenic fungi (12, 45). However, many species that were collected and described before the molecular era lack molecular data, which poses a significant challenge to their natural classification. Fresh collections of these species and clear morphological illustrations would aid tremendously in clarifying their naturally taxonomic placements, and these are currently being undertaken. Although many newly recorded and novel fungal species on invertebrates have been discovered, described and published across a plethora of journals, the published information is scattered and often not easily accessible. Moreover, the classification of entomopathogenic fungi is constantly updated, and these changes are hard to follow. Comprehensively compiling the taxonomic data concerning entomopathogenic fungi according to the original publications is in high demand by peers and researchers in related fields. An easily accessible and freely available platform would greatly facilitate the sharing and dissemination of knowledge among mycologists and other scientists. The online database is dedicated to gathering information from various peer-reviewed sources, allowing users to access up-to-date data in a time-saving and effective way. The website https://invertebratefungi.org/ was therefore established to systematically document data on invertebrate fungi in a web-based platform through which the end users can easily obtain access to relevant information.

**Table 1.** List of expert curators for the https://invertebratefungi.org/ website

Position	Name	Research field	Contact details
Head Curator	Yuan-Pin Xiao^a^	Entomopathogenic fungi	emmaypx@gmail.com
Managing Curator	De-Ping Wei^a^	Entomopathogenic fungi	elina19920601@gmail.com
Curator	Kevin D. Hyde^a^	Fungi	kdhyde3@gmail.com
Curator	Qing Tian^a^	Eurotiomycetes	tianqing124@gmail.com
Curator	Yuan-Bing Wang^b^	Entomopathogenic fungi	wangyb001@126.com

a Center of Excellence in Fungal Research, Mae Fah Luang University, Chiang Rai 57100, Thailand.

b The International Joint Research Center for Sustainable Utilization of Cordyceps Bioresources in China and Southeast Asia, Yunnan University, Kunming 650091, China.

### Invertebrate fungi website

The website contributes to the checklist of fungal genera and species associated with invertebrates. Current notes regarding taxonomic status, classification, host range and species with available morphological descriptions of fungal genera will be provided. For each genus, the species associated with invertebrate hosts will be presented and accompanied by Index Fungorum number, Facesoffungi number, descriptions, photographic plates, sequence data, phylogenetic data if available, known hosts and distributions, as well as other relevant information of importance. Systematic management and documentation of invertebrate-associated fungi will enhance the understanding of diversity and classification of invertebrate fungal pathogens.

### Construction, interface and visualization of database

**Figure 1.**
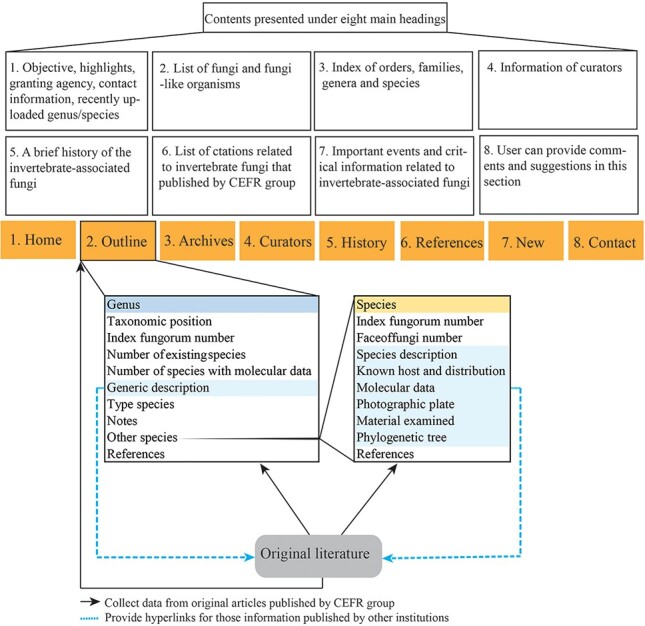
A schematic of the database structure.

**Figure 2.**
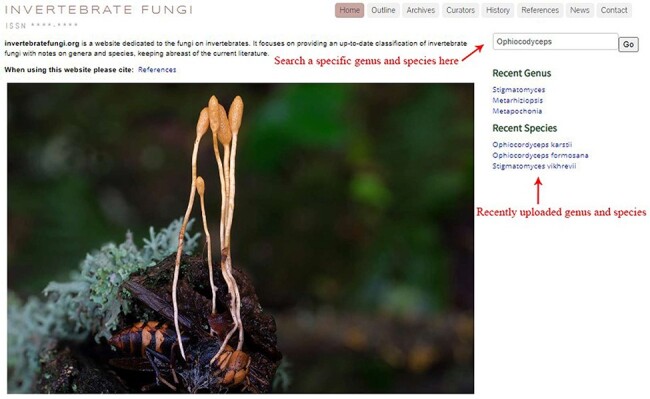
Top view of the homepage.

**Figure 3.**
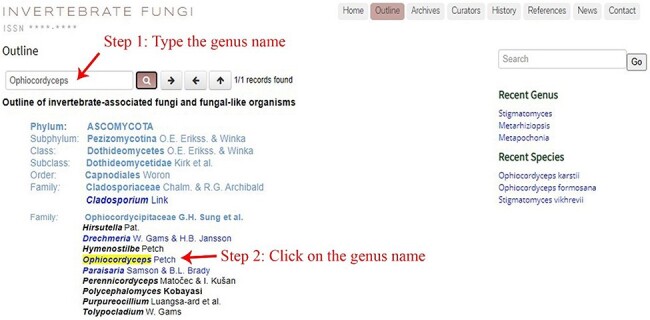
The outline of invertebrate-associated fungi and fungi-like organisms.

**Figure 4.**
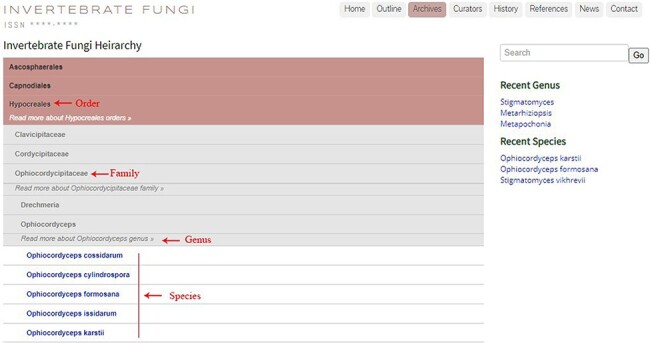
The ‘Archives’ section exhibiting fungal taxa in hierarchical order.

The online website, https://invertebratefungi.org/, allows end users to enter information on fungi associated with invertebrates via a friendly interface. This website comprises eight headings, each with its own distinct functionality (Figure 1). The research scope of this website is presented at the top-left corner of the homepage (Figure 2). The contact details and supporting grant information are located at the bottom of this page (Figure 2). The genera containing entomopathogenic species have been collected from peer-reviewed articles and systematically listed under the heading ‘Outline’ according to the most recent classification (Figure 3). Genera/species with available data will be automatically arranged in alphabetical order under the heading ‘Archives’ (Figure 4). Data of each genus and species presented on the website have been mainly extracted from original research articles published by the Center of Excellence in Fungal Research (CEFR) group. Data from collaborating institutes will also be included after getting permission. Otherwise, the taxonomic status of the genus will be noted and the entomopathogenic species will be listed along with a hyperlink to the original literature. Researchers who do not belong to the CEFR group also have the option to post the entomopathogenic species they discover via the managing curator of this website. The database will be updated continually to keep abreast of the current literature. Curators listed in the table 1 will take charge of collecting, managing, overseeing and reviewing the uploaded data in this webpage.

### How to use the website?

**Figure 5.**
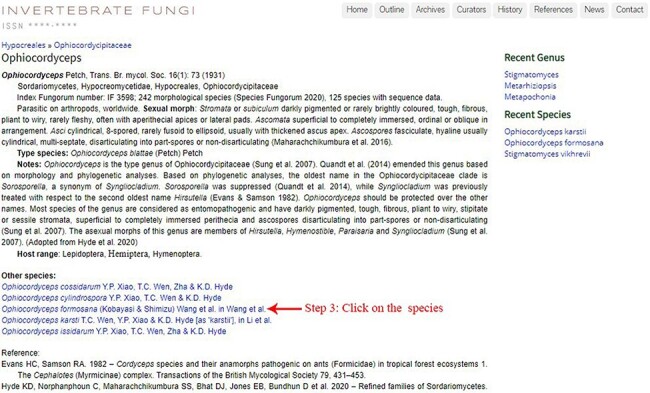
Information on the entomopathogenic genus *Ophiocordyceps*.

**Figure 6.**
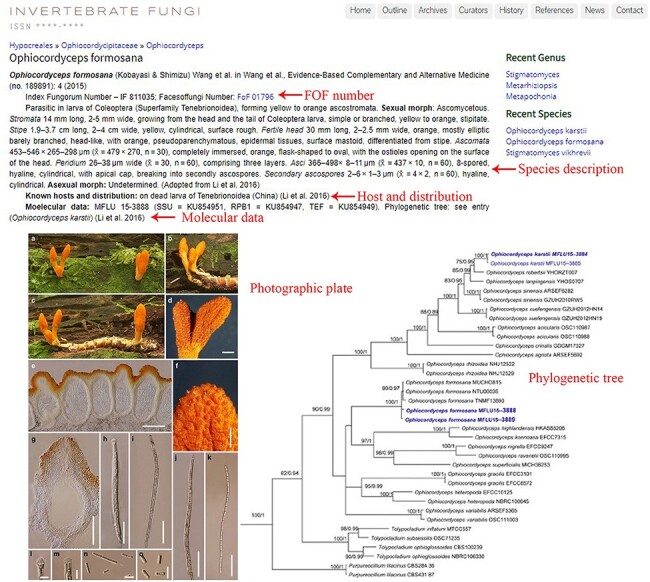
Information of the entomopathogenic species *Ophiocordyceps formosana*.

Instructions on searching a specific genus and species are illustrated below using the genus *Ophiocordyceps* and the species *O. formosana* as examples. Step 1: typing a specific genus name (e.g. *Ophiocordyceps*) into the search box located at the top-left corner of the ‘outline’ page allows users to find the genus in the outline (Figure 3). Step 2: a new window with information on *Ophiocordyceps* will pop up after clicking on the genus name (Figure 5). Step 3: the entomopathogenic species belonging to *Ophiocordyceps* will be presented beneath the ‘other species’ title, and users can find detailed information on a specific species (e.g. *O. formosana*) by clicking on the species name (Figures 5 and 6). The Facesoffungi Number (Figure 6) of a species is provided along with a hyperlink to the ‘Faces of fungi’ website (https://www.facesoffungi.org/).

Additionally, a search box is provided below the header bar, allowing the users to search the information on a specific genus and species by typing its name (Figure 2). Recently uploaded genera and species can be seen under ‘Recent Genus’ and ‘Recent Species’ tabs, respectively, which inform users of the newly added entries (Figure 2). Alternatively, clicking on the order name in the ‘archive’ page will result in a drop-down box appearing, where families of the selected order are displayed, and users can browse the genus and species composition of the selected family (Figure 4).

**Figure 7.**
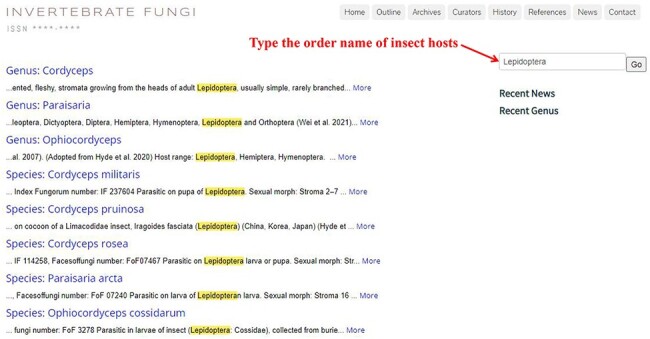
List of fungal species associated with Lepidopteran hosts.

Users can get a list of fungal species associated with specific hosts by typing the scientific or popular name of insect hosts in the search box below the eight header bars (see Figure 7).

## References

[1] May R.M. (1988) How many species are there on earth? *Science*, 241, 1441–1449. 17790039 10.1126/science.241.4872.1441

[2] Araújo J.P.M. and Hughes D.P. (2016) Diversity of entomopathogenic fungi. Which groups conquered the insect body? *Adv. Genet.*, 94, 1–39. 27131321 10.1016/bs.adgen.2016.01.001

[3] Samson R.A. , Evans H.C. and Latgé J.-P. (1988) Taxonomy of entomopathogenic fungi. In: *Atlas of Entomopathogenic Fungi*. Springer, Berlin, Heidelberg, pp. 5–16.

[4] Cooke M.C. (1892) *Vegetable Wasps and Plant Worms: A Popular History of Entomogenous Fungi, or Fungi Parasitic upon Insects*. Society for Promoting Christian Knowledge, London.

[5] Vega F.E. , Meyling N.V., Luangsa-ard J.J. et al. (2012) Fungal entomopathogens. *Insect Pathol.*, 2, 171–220.

[6] Mora M.A.E. , Castilho A.M.C. and Fraga M.E. (2017) Classification and infection mechanism of entomopathogenic fungi. *Arq. Inst. Biol. (Sao**Paulo)*, 84, 1–10.

[7] Gams W. and Zare R.A. (2003) Taxonomic review of the clavicipitaceous anamorphs parasitizing nematodes and other microinvertebrates. In: *Clavicipitalean Fungi*. pp. 26–81.

[8] Bench M.E. and White M.M. (2012) New species and first records of Trichomycetes from immature aquatic insects in Idaho. *Mycologia*, 104, 295–312. 21933923 10.3852/11-203

[9] Araújo J.P.M. , Evans H.C., Kepler R. et al. (2018) Zombie-ant fungi across continents: 15 new species and new combinations within *Ophiocordyceps*. I. Myrmecophilous hirsutelloid species. *Stud. Mycol.*, 90, 119–160. 29910522 10.1016/j.simyco.2017.12.002 PMC6002356

[10] Hyde K.D. , Tennakoon D.S., Jeewon R. et al. (2019) Fungal diversity notes 1036–1150: taxonomic and phylogenetic contributions on genera and species of fungal taxa. *Fungal Divers.*, 96, 1–242.

[11] Wei D.P. , Wanasinghe D.N., Hyde K.D. et al. (2020) *Ophiocordyceps tianshanensis* sp. nov. on ants from Tianshan mountains, R. P. China. *Phytotaxa*, 464, 277–292.

[12] Sung G.H. , Hywel-Jones N.L., Sung J.M. et al. (2007) Phylogenetic classification of Cordyceps and the clavicipitaceous fungi. *Stud. Mycol.*, 57, 5–59. 18490993 10.3114/sim.2007.57.01 PMC2104736

[13] Luangsa-ard J.J. , Tasanathai K., Mongkolsamrit S. et al. (2007) *Atlas of Invertebrate-pathogenic Fungi of Thailand, Vol 1*. BIOTEC, National Science and Tecnology Development Agency, Thailand.

[14] Kepler R.M. , Luangsa-Ard J.J., Hywel-Jones N.L. et al. (2017) A phylogenetically-based nomenclature for Cordycipitaceae (Hypocreales). *IMA Fungus*, 8, 335–353. 29242779 10.5598/imafungus.2017.08.02.08 PMC5729716

[15] Xiao Y.-P. , Hongsanan S., Hyde K.D. et al. (2019) Two new entomopathogenic species of *Ophiocordyceps* in Thailand. *MycoKeys*, 47, 53–74. 10.3897/mycokeys.47.29898 PMC6395454 30828254

[16] Hyde K.D. , Norphanphoun C., Maharachchikumbura S.S.N. et al. (2020) Refined families of Sordariomycetes. *Mycosphere*, 11, 305–1059.

[17] Hammond W. (1997) Laboulbeniales on beetles: host utilization patterns and species richness of the parasites. *Biodivers. Conserv.*, 6, 701–719.

[18] Tanada Y. and Kaya H.K. (1993) *Insect Pathology*. Academic Press. Inc. Harcourt Brace Avanovich, San Diego, pp. 359–361.

[19] Misra,J.K. and Horn,B.W. (eds). (2001) *Trichomycetes and Other Fungal Groups*. Science Publishers Inc, Enfield, New Hampshire, p. 396.

[20] Hibbett D.S. , Binder M., Bischoff J.F. et al. (2007) A higher-level phylogenetic classification of the Fungi. *Mycol. Res.*, 111, 509–547. 17572334 10.1016/j.mycres.2007.03.004

[21] James T.Y. , Kauff F., Schoch C.L. et al. (2006) Reconstructing the early evolution of fungi using a six-gene phylogeny. *Nature*, 443, 818–822. 17051209 10.1038/nature05110

[22] Kirk P.M. , Cannon P.F., Minter D.W. et al. (2008) *Stalpers, J.A. Ainsworth & Bisby’s Dictionary of the Fungi*. 10th edn. CAB International, Wallingford.

[23] Humber R.A. (2012) Entomophthoromycota: a new phylum and reclassification for entomophthoroid fungi. *Mycotaxon*, 120, 477–492.

[24] Spatafora J.W. , Chang Y., Benny G.L. et al. (2016) A phylum-level phylogenetic classification of zygomycete fungi based on genome-scale data. *Mycologia*, 108, 1028–1046. 27738200 10.3852/16-042 PMC6078412

[25] Tedersoo L. , Sánchez-Ramírez S., Kõljalg U. et al. (2018) High-level classification of the Fungi and a tool for evolutionary ecological analyses. *Fungal Divers.*, 90, 135–159.

[26] Wijayawardene N.N. , Pawłowska J., Letcher P.M. et al. (2018) Notes for genera: basal clades of Fungi (including Aphelidiomycota, Basidiobolomycota, Blastocladiomycota, Calcarisporiellomycota, Caulochytriomycota, Chytridiomycota, Entomophthoromycota, Glomeromycota, Kickxellomycota, Monoblepharomycota, Mortierellomy. *Fungal Divers.*, 92, 43–129.

[27] Martin W.W. (1981) *Couchia circumplexa*, a water mold parasitic in midge eggs. *Mycologia*, 73, 1143–1157.

[28] Mendoza L. , Vilela R. and Humber R.A. (2018) Taxonomic and phylogenetic analysis of the *Oomycota mosquito* larvae pathogen *Crypticola clavulifera*. *Fungal Biol.*, 122, 847–855. 30115318 10.1016/j.funbio.2018.04.010

[29] Vilela R. , Humber R.A., Taylor J.W. et al. (2019) Phylogenetic and physiological traits of oomycetes originally identified as *Lagenidium giganteum* from fly and mosquito larvae. *Mycologia*, 111, 408–422. 30985262 10.1080/00275514.2019.1589316

[30] Charnley A.K. Collins S.A. (2007) Entomopathogenic fungi and their role in pest control. In: Kubicek CP, Druzhinina, I. (eds.). *Environmental and Microbial Relationships: The Mycota IV*. 2nd edn. Springer-Verlag, Berlin Heidelberg, pp. 159–187.

[31] Boni S.B. , Mwashimaha R.A., Mlowe N. et al. (2021) Efficacy of indigenous entomopathogenic fungi against the black aphid, Aphis fabae Scopoli under controlled conditions in Tanzania. *Int. J. Trop. Insect Sci.*, 41, 1643–1651.

[32] Mudrončeková S. , Mazáň M., Nemčovič M. et al. (2021) Entomopathogenic fungus species *Beauveria bassiana* (Bals.) and *Metarhizium anisopliae* (Metsch.) used as mycoinsecticide effective in biological control of Ips typographus (L.). *J. Microbiol. Biotechnol. Food Sci.*, 2021, 2469–2472.

[33] Zhang L. , Fasoyin O.E., Molnár I. et al. (2020) Secondary metabolites from hypocrealean entomopathogenic fungi: novel bioactive compounds. *Nat. Prod. Rep.*, 37, 1181–1206. 32211639 10.1039/c9np00065h PMC7529686

[34] Maharachchikumbura S.S. , Chen Y., Ariyawansa H.A. et al. (2021) Integrative approaches for species delimitation in Ascomycota. *Fungal Divers.*, **109**, 155–179doi: 10.1007/s13225-021-00486-6.

[35] Raghoonundon B. , Zhao R.-L., He M.-Q. et al. (2021) www.basidio.org: an Online platform for the classification, identification and nomenclature of taxa within Basidiomycota. *Chiang Mai J. Sci.*, 48, 1186–1198.

[36] Wu N. , Dissanayake A.J., Manawasinghe I.S. et al. (2021) https://botryosphaeriales. org/, an online platform for up-to-date classification and account of taxa of Botryosphaeriales. *Database*, 2021, baab061. 10.1093/database/baab061 PMC8517499 34651182

[37] Pem D. , Hongsanan S., Doilom M. et al. (2019) https://www. dothideomycetes. org: an online taxonomic resource for the classification, identification, and nomenclature of Dothideomycetes. *Asian J. Mycol.*, 2, 287–297.

[38] Calabon M.S. , Hyde K.D., Jones E.B.G. et al. (2020) www. freshwaterfungi. org, an online platform for the taxonomic classification of freshwater fungi. *Asian J. Mycol.*, 3, 419–445.

[39] Jayawardena R.S. , McKenzie E.H.C., Chen Y.J. et al. (2019) https://onestopshopfungi. org/, a database to enhance identification of phytopathogenic genera. *Asian J. Mycol.*, 2, 281–286.

[40] Bundhun D. , Maharachchikumbura S.S.N., Jeewon R. et al. (2020) https://sordariomycetes. org/, a platform for the identification, ranking. *Asian J. Mycol.*, 3, 13–21.

[41] Kobayasi Y. (1941) The genus *Cordyceps* and its allies. *Rep. Tokyo Bunrika Daigaku Sect. B*, 5, 53–260.

[42] Kobayasi Y. (1982) Keys to the taxa of the genera *Cordyceps* and *Torrubiella*. *Trans. Mycol. Soc. Japan*, 23, 329–364.

[43] Mains E.B. (1958) North American entomogenous species of *Cordyceps*. *Mycologia*, 50, 169–222.

[44] Massee G. (1895) A revision of the genus *Cordyceps*. *Ann. Bot.*, 9, 1–44.

[45] Mongkolsamrit S. , Khonsanit A., Thanakitpipattana D. et al. (2020) Revisiting *Metarhizium* and the description of new species from Thailand. *Stud. Mycol.*, 95, 171–251. 32855740 10.1016/j.simyco.2020.04.001 PMC7426330

